# Scoping review of indicators and methods of measurement used to evaluate the impact of dog population management interventions

**DOI:** 10.1186/s12917-017-1051-2

**Published:** 2017-05-30

**Authors:** Elly Hiby, Kate Nattrass Atema, Rebecca Brimley, Alexandra Hammond-Seaman, Mark Jones, Andrew Rowan, Emelie Fogelberg, Mark Kennedy, Deepashree Balaram, Louis Nel, Sarah Cleaveland, Katie Hampson, Sunny Townsend, Tiziana Lembo, Nicola Rooney, Helen Rebecca Whay, Joy Pritchard, Jane Murray, Lisa van Dijk, Natalie Waran, Heather Bacon, Darryn Knobel, Lou Tasker, Chris Baker, Lex Hiby

**Affiliations:** 1 ICAM Coalition, c/o IFAW International HQ, Yarmouth Port, MA USA; 2 RSPCA International, Horsham, West Sussex UK; 3 Born Free Foundation, Horsham, UK; 4 HSI, NW Washington, D.C, USA; 5 World Animal Protection, London, UK; 6 Kennedy Animal Welfare Consultancy, Colchester, UK; 7 Global Alliance for Rabies Control, Kansas, USA; 8 Global Alliance for Rabies Control and Dept of Microbiology, NAS, University of Pretoria, Pretoria, South Africa; 9 The Boyd Orr Centre for Population and Ecosystem Health, Institute for Biodiversity, Animal Health & Comparative Medicine, University of Glasgow, Glasgow, UK; 10 Animal Welfare and Behaviour, School of Veterinary Sciences, University of Bristol, Langford, UK; 11 JMICAWE, Royal (Dick) School of Veterinary Studies, the University of Edinburgh, Roslin, UK; 12 Center for Conservation Medicine and Ecosystem Health, Ross University School of Veterinary Medicine, Basseterre, Saint Kitts and Nevis; 13 Independent animal welfare and behaviour consultant, Derbyshire, UK; 14 The Center for Animals and Public Policy, Cummings School of Veterinary Medicine at Tufts University, North Grafton, MA USA; 15 Conservation Research Ltd, Cambridge, UK

**Keywords:** Dog, Stray dog, Population management, Impact assessment, Indicators, Scoping review

## Abstract

**Background:**

Dogs are ubiquitous in human society and attempts to manage their populations are common to most countries. Managing dog populations is achieved through a range of interventions to suit the dog population dynamics and dog ownership characteristics of the location, with a number of potential impacts or goals in mind. Impact assessment provides the opportunity for interventions to identify areas of inefficiencies for improvement and build evidence of positive change.

**Methods:**

This scoping review collates 26 studies that have assessed the impacts of dog population management interventions.

**Results:**

It reports the use of 29 indicators of change under 8 categories of impact and describes variation in the methods used to measure these indicators.

**Conclusion:**

The relatively few published examples of impact assessment in dog population management suggest this field is in its infancy; however this review highlights those notable exceptions. By describing those indicators and methods of measurement that have been reported thus far, and apparent barriers to efficient assessment, this review aims to support and direct future impact assessment.

## Background

The global domestic dog population has been estimated to be over 700 million [1]. Reported ratios of dogs to humans vary from 91 dogs for every 100 people in the Philippines [2] to just 2 dogs for every 100 people in urban areas of Zambia [3], however for most populations the ratio is between 10 and 33 dogs per 100 people [4]*.* Maintaining dog population size and demography in balance with human ideals can be termed ‘dog population management’ with aims including reducing the number of unwanted dogs, keeping wanted dogs in a good state of health and welfare, and minimising risks presented by dogs to public health and other animals. An example of a problem targeted by dog population management is reducing the euthanasia of unwanted dogs; in the USA an estimated 3 million dogs and cats are euthanised in shelters each year [5]. Public health problems targeted include dog bites and rabies; an estimated 74,000 people die of rabies annually [6] and over 99% of human deaths from rabies involve transmission of the virus from dogs [7]. In the USA, 4.5 million people are bitten by dogs each year, an annual incidence of 1500 bites per 100,000 people, with one in five of these incidents requires medical attention for the bite [8].

In some countries, the vast majority of domestic dogs are confined and are provided with resources directly by their owners, while in others, owned dogs may be unconfined and free to roam, resulting in a visible roaming dog population comprising both owned and unowned dogs. Further, the definition of ownership may vary with country, from a fully confined pet that resides mainly in the home to a dog that lives exclusively outdoors and receives care from more than one household, sometimes termed a ‘community dog’. Interventions to manage dog populations can take many forms, designed according to the dog population dynamics, disease risks and dog ownership practices of the location. Interventions include activities such as sterilisation, basic veterinary care (e.g. vaccination and deworming), rehoming of dogs and education of dog owners. Regardless of the intervention used, there is a need to assess the effectiveness by measuring changes in relevant indicators.

Measuring indicators of impact can reveal whether or not an intervention leads to anticipated changes and can provide valuable learnings on intervention design and implementation. Such learnings can facilitate incremental improvements to the intervention, and when these learnings are disseminated can also inspire changes in other interventions. Evaluation of impact may also be required by funders of interventions, including government agencies (in the case of interventions funded by public money) and private or organisational donors, commonly associated with interventions run by non-governmental organisations.

This scoping review describes efforts to measure indicators of dog population management effectiveness, also known as impact assessments. Mays et al. [9] define scoping reviews as “to map rapidly the key concepts underpinning a research area and the main sources and types of evidence available, and can be undertaken as standalone projects in their own right, especially where an area is complex or has not been reviewed comprehensively before”. The goal of this scoping review is to describe and critically appraise previously used and potential indicators for further consideration and use in future assessments of dog population management effectiveness. The review also provides key learning points relevant for such impact assessments. The results of this scoping review contributed to development of guidance for practical implementation of impact assessments in dog population management interventions [10].

## Method: Literature review

### Search strategy

The literature search covered published peer-reviewed journal articles, conference presentations, reports published by authors but not subject to peer-review, and personal communications with researchers and dog population intervention managers. For the purposes of this search, dog population management was defined as an intervention that targets either, or both, the currently unowned dog population or the owned dog population assumed to be a potential source of unowned or unwanted dogs. The purpose of the intervention would include reduction in unowned and unwanted dogs or in zoonotic disease risk presented by the dog population, but would likely have other impacts in mind. The activities involved in the intervention would vary, but would likely include sterilisation, basic veterinary care such as vaccination, education of owners and rehoming.

The search was performed using the following methods:

- Review of all presentations at the 1st International Dog Population Management conference in 2012 [11], followed by review of any cited articles or reports mentioned in those presentations relating to assessment of effectiveness.
- Dog population management experts from the International Companion Animal Management Coalition (a coalition that includes some of the largest charities currently investing in dog population management internationally), were asked to provide relevant publications, reports and presentations.
- Online search: PubMed, Science Direct (including using their ‘recommended articles’ function) and Google Scholar were searched for papers with (dog OR canine) AND (control OR management OR population). These limited search terms were used to focus the search on literature relating to dog population management, as defined previously in this section, as opposed to the larger body of literature exploring changes in owned dog health and behaviour over time.
- Snowballing: following relevant citations mentioned in identified papers for review.
- In addition, interviews (email, phone or in person) were conducted with authors of particularly relevant publications and reports and with managers of interventions known to have invested in assessing their effectiveness. This was done to establish detailed understanding of the indicators and methods of assessment used.

The literature search was completed by end of March 2015, with website links updated in late 2015. However, 2 conference presentations that were included in the scoping review were subsequently published in 2016, the references were therefore replaced with the peer-reviewed journal references, as the relevant content from the conference presentations had been retained.

### Inclusion criteria

As this review was focused on which indicators could be used for assessing change resulting from an intervention, a criterion for inclusion was that an attempt was made to measure an indicator of intervention *impact*. Impact was defined as something the intervention aimed to *change*. This can be contrasted with indicators of intervention *effort*, which reflect the time and resources put into implementing the intervention. An example of an indicator of impact would be the proportion of the roaming dog population with a body condition score of 2 (thin) or 1 (emaciated); an example of an indicator of effort would be the number of dogs sterilised and vaccinated by an intervention.

The review included all studies that involved repeated measurement of an impact indicator, allowing for an assessment of how this indicator had changed over time and therefore potentially reflecting intervention effectiveness, if an intervention had taken place over the same time period. However, due to the relative scarcity of reports of indicator use in the literature, studies that reported only a single measurement of an impact indicator, reflecting a baseline for that indicator against which change could be measured over time, were also reviewed.

A further criterion for inclusion was that the literature was available in English.

### Ethics and consent to participate

This review did not involve primary research with either people or non-human animals, hence no ethical approval or consent to participate was required.

## Results and Discussion

### Literature characteristics

The scoping review encompassed 120 items of literature in total; 26 were found to report the impact of a dog intervention by measuring one or more indicators (Table 1). Of these 26 impact assessments, four were presented at conferences, 19 were reported in peer-reviewed literature, 2 were in reports and 1 book. Much of the remaining literature explored the demography and interactions of dogs with other species, including humans, but only reported measurement at a single point in time or if changes were followed over time, this was not in response to an intervention. Nevertheless, some of these studies are described in this review as they provided details on indicators that could be used for future impact assessment.

**Table 1:** Summary of the 26 items of literature reviewed that reported a change in one or more impacts following a dog population management intervention

Impact assessed	Indicator(s) used	Change in indicator following intervention?	Method of measurement	Study design type	Intervention type (limited to dog-related activities)	Country (city or region if applicable), Continent	Publication type	Reference
1. Improve dog welfare	Body condition score	No	Repeated clinical exam of cohort of dogs	Quasi-experimental; prospective cohort	Rabies vaccination	Tanzania, Africa	Peer-reviewed publication	[17]
1. Improve dog welfare	Body condition score	Yes	Clinical exam of dogs whilst in intervention clinic or during handling for vaccination (control group)	Observational; retrospective cross-sectional	Neutering, vaccination and return of roaming dogs	India (Rajasthan), Asia	Peer-reviewed publication	[15]
	Presence of ticks/fleas	Yes						
	Open wounds	Yes						
	Antibodies to canine infectious diseases (serology)	Yes						
1. Improve dog welfare	Body condition score	Yes	Street surveys of roaming dogs	Quasi-experimental; Cross-sectional	Neutering, vaccination and return of roaming dogs	India (Jodhpur), Asia	Peer-reviewed publication	[16]
	Skin condition	Yes	Clinical exam of dogs whilst in intervention clinic					
	Open wounds	No						
1. Improve dog welfare	Dog-dog aggression	No	Video surveillance of roaming dogs	Experimental; prospective cohort	Castration of male dogs	Chile (Puerto Natales), S America	Peer-reviewed publication	[22]
	Dog-human aggression	No						
	Interspecies aggression	No						
1. Improve dog welfare	Body condition score	Yes	Clinical exam of dogs whilst in intervention clinic	Observational, no control group; repeated cross-sectional	Neutering, rehoming, basic vet care, euthanasia	USA (Lakota Reservation), N America	Conference presentation	[14]
	Skin condition	Yes						
2. Improve care provide to dogs	Proportion of dogs brought to clinic, as opposed to needing to be caught	Yes	Interview of local grocery stores					
	Dog food purchases	Yes						
1. Improve dog welfare	Body condition score	Yes	Street surveys of roaming dogs	Quasi-experimental; repeated cross-sectional	Neutering, rabies vaccination, basic vet care, bite prevention education	Sri Lanka (Colombo), Asia	Conference presentation	[13]
	Skin condition	Yes						
3. Reduce dog density/stabilise turnover	Number of dogs observed in sample of wards	Yes						
	Percentage of lactating females	Yes						
3. Reduce dog density/stabilise turnover	Number of dogs observed in ‘zones’ demarked by intervention	Yes	Mark (ear notch applied during intervention)-resight survey of roaming dogs	Observational; repeated cross-sectional	Neutering, vaccination and return of roaming dogs	India (Jaipur), Asia	Conference presentation	[42]
3. Reduce dog density/stabilise turnover	Number of dogs in sample areas	Yes	Mark (paint applied during survey)-resight survey of roaming dogs	Quasi-experimental; Cross-sectional	Neutering, vaccination and return of roaming dogs	India (Jodhpur), Asia	Peer-reviewed publication	[43]
3. Reduce dog density/stabilise turnover	Number of dogs per square mile of sampled areas	No	Mark (individual dogs identified and recorded using photographs)-resight survey of roaming dogs, also known as photo capture-recapture	Observational, no control group; repeated cross-sectional	Roaming dogs removed by Animal Control and housed for returning, rehoming or euthanasia in a local government pound	USA (Baltimore), N America	Book	[48]
3. Reduce dog density/stabilise turnover	Percentage of lactating females and puppies	Yes	Street surveys of roaming dogs	Observational; repeated cross-sectional	Neutering, vaccination and return of roaming dogs	Nepal, Asia	Conference presentation	[51]
					Rabies vaccination			
	Male:female	No						
3. Reduce dog density/stabilise turnover	Percentage of households experiencing dog mortality in past 12 months	Yes	Questionnaire of dog owners	Observational; cross-sectional	Neutering, vaccination and basic health care for owned and roaming dogs	Thailand (Kho Tao), Asia	Report	[27]
	Percentage of owned dogs adopted	Yes						
3. Reduce dog density/stabilise turnover	Number of dogs observed on 6 standard routes	Yes	Street surveys of roaming dogs	Observational; repeated cross-sectional	Neutering, vaccination and return of roaming dogs	India (Jaipur), Asia	Peer-reviewed publication	[41]
4. Reduce risks to public health	Human rabies cases	Yes	Data collected from local hospital	Quasi-experimental; cross-sectional				
4. Reduce risks to public health	Reported dog bites from local hospital	Yes	Accessed publically available hospital reports	Observational; repeated cross-sectional	Neutering, vaccination and return of roaming dogs	India (Jaipur), Asia	Peer-reviewed publication	[57]
4. Reduce risks to public health	Human bite injuries from suspect rabid dogs	Yes	Animal-bite injury data collected from Government District Hospitals	Experimental; repeated cross-sectional	Rabies vaccination	Tanzania, Africa	Peer-reviewed publication	[58]
4. Reduce risks to public health	Dog rabies cases	Yes	Data collected from district Veterinary and Health authorities	Observational; repeated cross-sectional	Rabies vaccination	Indonesia (Bali), Asia	Peer-reviewed publication	[60]
	Dog bite injuries treated with PEP	Yes						
	Human rabies cases	Yes						
4. Reduce risks to public health	Human rabies cases	Yes	Data collected from Peruvian Ministry of Health	Observational; repeated cross-sectional	Rabies vaccination	Peru (Lima), S America	Peer-reviewed publication	[65]
	Dog rabies cases	Yes						
4. Reduce risks to public health	Incidence of livestock with hydatid cysts	Yes	Surveillance of offal at slaughter houses	Observational; repeated cross-sectional	Dog deworming	New Zealand, Australasia	Report	[68]
4. Reduce risks to public health	Surgical incidence of cysts in humans	Yes	Quarterly reports from all hospitals	Observational; repeated cross-sectional	Dog deworming	Australia (Tasmania), Australasia	Peer-reviewed publication	[69]
	Incidence of hydatid cysts in sheep	Yes	Surveillance of offal at slaughter houses					
	Incidence of infected dogs	Yes	Presence of worms following purging of dogs					
4. Reduce risks to public health	Incidence of hydatid cysts in sheep	Yes	Surveillance of offal at slaughter houses	Observational; repeated cross-sectional	Dog deworming	Falkland Islands, S America	Peer-reviewed publication	[70]
	Incidence of infected dogs	Yes	ELISA test for serum antibodies and ELISA test for copro-antigens					
4. Reduce risks to public health	Incidence of human cases of leishmaniasis	Yes	Data collected from State Epidemiological Surveillance Centre	Observational; repeated cross-sectional	Dog culling	Brazil (Aracatuba), S America	Peer-reviewed publication	[71]
4. Reduce risksto public health	Incidence of human cases of leishmaniasis	Yes	Data collected from Zoonoses Control Centers (dog seropositive status tested by ELISA and confirmed by Indirect Immunofluorescency test)	Observational; repeated cross-sectional	Dog vaccination and culling	Brazil (Aracatuba and Belo Horizonte), S America	Peer-reviewed publication	[73]
	Incidence of leishmaniasis infection in dogs	Yes						
4. Reduce risks to public health	Incidence of human infection with leishmaniasis	Yes	LST conversion and DAT of finger-prick blood samples from children	Experimental, cluster randomized control trial; prospective cohort	Deltamethrin-impregnated dog collars	Iran (Kalaybar and Meshkin-Shahr), Middle East	Peer-reviewed publication	[74]
	Incidence of leishmaniasis infection in dogs	Yes	DAT of dog serological samples					
4. Reduce risks to public health	Incidence of leishmaniasis infection in dogs	Yes	Antibody test - rK39 dipstick of serological samples Parasitology tests - Examination of lymph-node smears and PCR of dermal tissue	Experimental parallel-group randomized control trial; prospective cohort	Repellent and insecticidal (imidacloprid 10%/permethrin 50%) spot-on for dogs	Italy, Europe	Peer-reviewed publication	[75]
4. Reduce risks to public health	Dog rabies cases	Yes	Data collected from municipal vet authority	Observational; repeated cross-sectional	Neutering, rabies vaccination, basic vet care, bite prevention education	Sri Lanka (Colombo), Asia	Peer-reviewed publication	[59]
	Dog bite injuries treated with PEP	Yes						
5. Improve public perception	Summative acceptance score	Yes	Data collected from bite centre in General Hospital					
	Number of dog-related problems	Yes	Attitude statements in questionnaire					
			Participatory research with focus groups					
6. Improve rehoming/adoption centre performance	Intake rates	Yes	Monthly reporting of data from each of six rehoming/adoption centres	Observational; prospective cohort study	Mixed – each of 6 communities selected the most locally relevant intervention. Examples included high-volume spay/neuter services, adoption promotions, new fund-raising strategies and community engagement	USA, N America	Peer-reviewed publication	[78]
	Live release rates	Yes						
6. Improve rehoming/adoption centre performance	Intake rates	No	Reporting of intake data from all rehoming/adoption centres involved in five Maddie’s Fund (donor) community programs	Observational; retrospective cohort study	Low cost neutering for owned dogs	USA, N America	Peer-reviewed publication	[79]

Definitions of terms used in ‘Study design’ column:

*Observational* studies are usually those where no intervention was used. The studies included in this table are all related to assessing the impact of an intervention. However in some studies, measurements were taken from dogs prior to them being intervened upon, e.g. taking body condition scores as they arrive at the intervention clinic, to assess retrospectively if they have benefited from living in a location where an intervention has been used with other dogs; these studies are defined as observational because none of the dogs have directly undergone treatment at the time of observation

*Experimental* studies are those where dogs that had experienced intervention were compared to those that had not (a control group) and where allocation to intervention or control was done randomly (includes randomised control trials). Randomisation can be done where each dog is randomly assigned to be part of the intervention or control; this is called *parallel-group*. Or where groups of dogs, such as those living in particular villages, are randomly assigned to the intervention or control; called *cluster* randomisation

*Quasi-experimental* studies are those where dogs that had experienced intervention were compared to those that had not (a control group) but allocation to intervention or control was not done randomly, e.g. owners brought their dogs or catchers caught whichever dogs were accessible

*Repeated cross-sectional* studies have observed a sample of dogs from the same population on two or more occasions, but it is not known if the same dogs appear in each sample, e.g. observing all the dogs visible along a survey route before the intervention started and again observing all dogs visible along the same route after the intervention has been running for a period of time, some of those dogs will be in both surveys, others will only be seen in one. This may also be termed a between-subject design

*Longitudinal studies* observe the same individual dogs at two or more points over time, also termed within-subject design. When these include both dogs that have been part of an intervention, and those in a control group, it is termed a *cohort* study

*Retrospective studies* (Latin derived prefix, “retro” meaning “back, behind”) look back at the history of a sample of dogs to see if differences between these dogs can be explained by what has happened to them in the past, usually comparing whether they have been part of an intervention or not

*Prospective studies* (Greek derived prefix, “pro” meaning “before, in front of”) start with a sample of dogs and measures what happens to them over time to examine causal associations, when part of a cohort study this will involve some of the dogs being subject to the intervention and others being part of a control group

The methods of measurement described within the literature reviewed were varied and often several methods were employed within one study. The methods included questionnaires (commonly structured as knowledge, attitude and practice (KAP) surveys), participatory research methods [12], street dog surveys, health assessments of dogs recruited to an intervention and analysis of data from secondary sources.

### Impact assessment

The literature reported measurement of 29 indicators for assessing the impact of a dog population management intervention. Through consultation with members of the International Companion Animal Management Coalition and other dog population management experts, a list of eight commonly stated impacts of interventions was developed. The 29 indicators identified by the scoping review could then be categorised into the following eight impacts, although not equally distributed, with some impacts measured by a greater number of indicators: 1) improve dog welfare; 2) improve care provided to dogs; 3) reduce dog population density or stabilise population turnover; 4) reduce risks to public health; 5) improve public perception; 6) improve rehoming centre performance; 7) reduce negative impact of dogs on wildlife and 8) reduce negative impact of dogs on livestock.

#### Impact 1: Improve dog welfare

The following are animal-based indicators for measuring dog welfare, these can be split into those focusing on physical health and those aimed at identifying behavioural signs of psychological wellbeing.

##### Physical health indicators of dog welfare

Physical health was most commonly assessed through measurement of **body condition score.** Body condition score was found to increase following intervention in all cases where it was used in impact assessment of an intervention [13–16], except in response to a rabies vaccination only intervention where no change was seen [17]. Body condition scales used were either the 9-point Purina scale validated by Laflamme [18] or a simplified 5-point version of this scale.

The presence of a visible **skin condition** was also used as an indicator of physical health and was found to change following intervention on three occasions where it was used in evaluation [13, 14, 16]. This was usually measured as simply presence or absence of a visible skin condition without any attempt at further diagnosis, with any sign of hair loss or scaly/sore skin counted as a skin condition. Steinberger [14] also describes a reduction in ‘serious mange’ defined as ‘large areas affected and/or bleeding’, suggesting that they used more than two categories or a scale of severity.

Related to skin conditions was the presence of **external parasites: fleas and ticks**. This could not be measured using observation at a distance and instead was measured via clinical examination as dogs passed through the intervention in one study in India [15]. Presence or absence of external parasites was used rather than any measure of infestation severity. A greater proportion of the dogs were found with ticks in two cities undergoing ABC interventions (ABC is an acronym for Animal Birth Control and involves catching, sterilising, vaccinating and then returning stray dogs) as compared to one city with no intervention; although the incidence of tick-borne disease (ehrlichiosis) was lower in ABC cities suggesting other factors were important for this disease.

The presence of **open wounds** recorded during clinical examination was also used in evaluating ABC interventions in two studies [15, 16]. Yoak et al. [15] found significantly fewer dogs with open wounds in ABC cities while Totton et al. [16] found no significant change within one city following ABC intervention. Yoak et al. [15] suggests that open wounds are a sensitive indicator of welfare during the breeding season as injuries were observed following fights between dogs around females in oestrus, which were less frequent in the ABC cities.

The **occurrence of canine infectious diseases**, other than rabies, was rarely reported. Yoak et al. [15] utilised blood samples taken from dogs as they passed through ABC interventions in Indian cities, i.e. blood was drawn when the dog was already anesthetised for surgical neutering, and also from a sample of dogs caught in a non-ABC city that had blood drawn as they were vaccinated against rabies. From these blood samples they tested for antibodies to several infectious diseases, assuming that antibody titres would be reflective of infection rather than vaccination, given that vaccination against these diseases is very rare in Indian street dogs. *Leptospira interrogans serovars, Ehrlichias canis* and Infectious Canine Hepatitis (ICH) were found in significantly lower proportions of dogs in ABC cities, whilst seroprevalence to canine distemper virus, canine parvovirus and *Brucella. canis* did not differ significantly between ABC cities and the city with no intervention.

##### Behavioural indicators of dog welfare

Animal welfare is a function of both physical health and psychological wellbeing [19], hence behavioural indicators measure relevant components not captured through physical indicators alone. Behavioural indicators of welfare have been used in several species and are arguably a better measure of emotional state, and therefore the animal’s perception of its own welfare, than physical indicators. In addition, some behaviours may not only be an indicator of negative emotional state but may cause welfare problems themselves, for example aggression between dogs.

Although behaviour has been used to measure changes in the welfare state of individual dogs in kennels (e.g. [20, 21]), we found only one example of measuring change in behaviour in roaming dog populations in response to dog population management interventions [22]. In Chile, Garde et al. [22] measured dog-dog aggression, dog-human aggression and inter-species aggression in roaming dogs. There was no clear impact of the intervention (castration of males dogs) on behaviour.

Pal et al. [23] also measured agonistic behaviour in roaming dogs in India and found peaks in aggression between dogs during periods of oestrus and lactation. While it might be hypothesized that aggression between dogs would be reduced by interventions that reduce oestrus and lactation events through control of female reproduction, no impact of intervention was tested by this study. In contrast to aggression, the presence of behaviours that reflect positive mental states could be used as a measure of good welfare, including amicable social behaviours such as allogrooming and play. **Play behaviour** in dogs has been used as a measure of welfare in kennelled dogs [24] and has been observed in young street dogs in India [25], no literature was found on whether this behaviour changes with an intervention.

The behaviour of dogs during **interactions with people** has been studied in pet dogs and has been shown to reflect previous interactions, with less play behaviour with owners and fewer approaches to new people performed by dogs reported to have been trained using punishment [26]. The response of street dogs to trained handlers is being assessed in Jamshedpur, India (Joy Lee *pers. comm*). Dogs are scored according to their response to an attempt to pick them up by hand, on a scale of 0–5 with 0 being an immediate aggressive response to the handler’s presence and 5 being able to pick up the dog easily. A negative response to a person is assumed to reflect a negative emotional state and/or a past negative experience with people. This measure has not yet been tested for evaluation of intervention impact and is currently used only as a way of planning intervention implementation, in estimating of the number of dogs that can be picked up for neutering. As described previously, interaction between dogs and people on the street was used in Chile [22] but not found to differ with the intervention.

#### Impact 2: Improve care provided to dogs

For some interventions the desired impact will be an increase in the quality of care provided to dogs and/or encourage ‘responsible dog ownership’; responsible dog ownership may be defined as good care provided to dogs, efforts to reduce the risks that dogs present to other animals, people, the environment, and contributing to good population management by not abandoning dogs and rehoming through adoption. An improved quality of care and increased responsibility measured by resource-based indicators is likely to be paired with actual dog welfare improvement, which could be captured by animal-based indicators.

A change in the **rate of acquisition of dogs,** and in particular **the proportion of dogs that were adopted from the street,** was reported by the impact assessment conducted on the intervention run by the organisation ‘Noistar’ on Kho Tao, Thailand [27]. They found a steep increase, from 28% to 64%, of owned dogs reported as adopted following an 18-month period of intervention (Natasha Lee, *pers. comm*).

One novel indirect indicator of care provided to dogs was an **increase in the purchase of pet food** from local grocers in a Lakota Reservation in the USA, during an intervention that combined spaying/neutering, off-site adoption and basic health care provisioning [14]. A decline in number of dogs on the reservation and a visible improvement in body condition and skin condition were observed. It was unclear if the improvement was due to the proactive efforts of the owners or it was the passive effect of a reported reduction in dog density. Change in sales of commercial dog food was then explored with local grocers, exposing an increase in sales of commercial dog food despite a reduction in the overall dog population size.

A further indicator of care or ‘responsibility’ by owners is **owner engagement in the intervention** itself. In the Lakota reservation, the proportion of dogs voluntarily brought to clinics rather than caught and transported by project staff increased over time, including dogs that were already spayed/neutered, but were brought back for vaccination and ‘wellness examinations’ [28].

The **performance of specific dog-care practices** was almost always measured through a questionnaire, either delivered as a face-to-face interview (e.g. [29, 30]) or over the phone (e.g. [31]). No examples of repeated measures over time in order to assess the impact of an intervention on care practices were found. Questionnaires require significant resources to implement and analyse, a potentially more efficient alternative to questionnaires are participatory research methods [12]. These approaches utilise groups of local people lead by a facilitator to discuss, measure and report on important data, usually benefiting from exercises that encourage engagement of all members of the group. Their use with dogs has been reported once in the literature, where a set of participatory exercises was used to explore feeding of owned dogs [32]. Similar approaches have been used to explore and improve the care provided to working equines by the charity ‘The Brooke’ [33].

#### Impact 3: Reduce dog population density/stabilise population turnover

Impacting on dog population numbers is a commonly stated goal of dog population management. This may be defined as reducing population size or density, often of a sub-population of dogs such as roaming or unowned dogs, or as stabilising population turnover (i.e. reducing births and deaths, with each animal living longer on average).

##### Reducing dog population density or size

Indicators of population size may be **estimates of absolute size** (for example the number of owned dogs living within in a city boundary) or **relative indices of dog density** (for example the number of roaming dogs observed along a set of survey routes). Methods of estimating dog population size include household questionnaires to estimate owned dog populations and street surveys to estimate roaming dog populations.

**Questionnaires** quantifying both the number of dogs and people living in each household were used to establish an owned **dog:human ratio**, which when applied to human census data, provides estimates of the **absolute size of the owned dog population for defined areas** [30, 34]. Other studies do not extrapolate a dog population estimate but just provide the dog:human ratio as a relative measure of dog density [35, 36]. A potentially more intuitive way of presenting this ratio would be as the number of dogs per 100 people, where a larger number indicates more dogs, as opposed to the 1 dog:*X* humans where a larger *X* means fewer dogs. Two studies measured the physical area of the study site and used this in combination with the total number of dogs found through the questionnaire to calculate the **number of owned dogs per km** ^**2**^ [30, 37].

No studies cited thus far for dog population size used questionnaires repeatedly to explore the impact of an intervention. Instead, they used questionnaires on a single occasion to obtain data on dogs and the geography and socioeconomic status. One exception was Kitala et al. [38] who used questionnaire surveys on two occasions one year apart to estimate birth and death rates, and therefore population turnover, in preparation for planning a rabies vaccination campaign. However, this approach was used to investigate a population ahead of an intervention as opposed to measuring its impact. Other exceptions include cohort studies that used repeated questionnaires to track a group of dogs over time (including their movements to different households) or all dogs living in a sample of households. Morters (South Africa and Bali; [39] and Knobel (South Africa; Darryn Knobel, *pers. comm*) have been using this approach to track changes occurring in natural populations without intervention, whilst Czupryna et al. [40] in Tanzania and Baker in Guatemala (Chris Baker, *pers. comm*) monitored cohorts during intervention.

Questionnaires can provide a breadth of information on dog ownership but are relatively time consuming to design, implement and interpret, which may explain why this methodology has been used more commonly for in-depth initial assessment and less frequently for regular monitoring of dog populations.

In comparison, **street surveys** of roaming dogs are more frequently used to evaluate the impact of an intervention [13, 41–43]. The methods of measurement used in these street surveys falls into three categories:

- Several studies used **mark-resight** to estimate the **absolute size of the roaming dog population for a defined area.** This approach usually used resighting of marks applied as part of the intervention, such as ear notches applied during anaesthesia for ABC [42, 44] or collars fitted during a vaccination campaign ([34, 45]; these are not impact assessment studies, i.e. they did not repeat resight surveys over time to establish a change in dog density in response to an intervention). Other studies used naturally occurring marks and scars to identify a proportion of the individuals [46, 47], recording these individuals using photographs and then following with resight surveys; these studies did not assess a change in dog population size resulting from an intervention. Two studies did repeat mark-resight surveys at different points in time to assess a change in population size: Totton et al. [43] applied paint spray marks as part of the survey process, with resight of marked dogs occurring on subsequent days, on two occasions approximately 1.5 years apart, in the Indian city of Jodhpur. In the 1970s, Beck [48] utilised photographs to identify all the dogs in his study area of Baltimore, USA, describing them as “easily distinguishable as individuals”; allowing him to conduct multiple resight surveys, on two occasions 1 year apart, to estimate changes in the absolute size of the roaming dog population over time. Although this study was published in 1973, a recent test of this method using population simulations suggested it was a robust method for estimating population size so long as underlying assumptions were not severely violated [49].
- One study reported the use of **direct observation on set routes** for establishing a relative measure of dog numbers, specifically the **total number of roaming dogs seen on six routes** [41, 50]. By repeating the observation over time, the change in the number of dogs seen on these routes was presumed to reflect the change in the wider dog population density.
- Sankey et al. [13] used direct observation during exhaustive searches of plots (plots were local administrative areas or ‘wards’). These were repeated over time providing a measure of the change in the **number of roaming dogs seen per plot**.

Street surveys require fewer resources to implement than questionnaires which may explain why they are more commonly used for evaluation. In addition, by their very nature, they measure the roaming dog population as opposed to the confined dog population; the former is usually the population of interest for dog population management.

The methods of conducting street surveys differ in both their ability to estimate absolute population size versus providing a measure of relative density and also in the time taken to conduct the survey. These two factors are linked; when absolute population size is desired, the survey needs to be more intensive to allow the use of methods such as mark-resight, exhaustive searches of plots and questionnaires where owners are asked about dog confinement versus roaming. The need for an indicator of absolute size should be carefully considered because the additional resources required may reduce the number of times a measure is taken or the size of survey area covered, therefore limiting the ability of the sample to represent the wider intervention area. An example of this is illustrated by two cities in Rajasthan, India; in Jaipur, a set of six standard survey routes were repeated at the same time of day and year, these were completed in approximately 12 h spread over six days [50]; this can be compared to the six areas used for mark-resight in Jodhpur where each of the six areas required approximately 10 h over five days, leading to 60 h in total [43]. The mark-resight approach therefore took five times longer to complete than the set of standard survey routes approach. This may partially explain why the less intensive Jaipur survey has been used at least once per year for 16 years, yielding a unique long-term dataset on dog density in response to an intervention, whilst the mark-resight survey was only conducted twice in Jodhpur.

##### Stabilise dog population turnover

In some cases, reducing dog population size may not be desired by local communities, however reducing turnover may be a goal. A population with high turnover has high birth and mortality rates, with each dog having on average a relatively short lifespan; the high mortality rate implies high morbidity and usually a poor state of welfare. High population turnover is also undesirable during vaccination campaigns as vaccinated dogs die and puppies are born unvaccinated, reducing herd immunity. The following indicators were reported in the literature as reflecting changes in turnover.

A change in the **percentage of lactating females** and **of puppies** seen during street surveys were reported following a period of intervention [13, 51]. It can be argued that these indicators of breeding activity would be the first to be seen ahead of any change in dog population size, as changes in population size would only occur as dogs die and are not replaced at the pre-intervention rates.

An estimate of **mortality** and **fecundity** (average birth rates per female) was reported in several studies where repeated measures were used. One very simple indicator of mortality is the percentage of dog-owning households that report a dog dying in the previous 12 months. A decrease in mortality was reported following 18 months of intervention on the island of Kho Tao, Thailand [27]. Cohort studies used repeated investigations of a group of dogs to obtain reliable birth and death rates ([39, 40] Darryn Knobel *pers. comm*, Chris Baker *pers. comm*), although only the populations studied by Czupryana et al. [40] and Baker were under intervention. Totton et al. [43] fitted a demographic model to the data collected through street surveys, investigation of uteri during spaying and estimates of pregnancy [52] to calculate mortality and fecundity for dog populations in the city of Jodhpur, India.

One final indicator with the potential for measuring population stability and also welfare is the **ratio of males:females**. This ratio was commonly reported in the owned dog demography studies reviewed, usually with males predominating [30, 34, 37]. However in two urban locations in Rajasthan, India, where the roaming dog population is assumed to be predominately unowned, the sex ratio is equal or close to equal [41, 43]. The assumption is that, on average, litters are born with an equal number of males and females, but there is disproportionate mortality in females potentially due to preferential adoption and care of males and purposeful killing of females in the owned dog population. One example of disproportionate mortality in females was a greater number of females being sold to meat traders in Bali as compared to males [39]. Where higher mortality in females is due to a problem of unwanted litters, an intervention that includes spaying of females has an opportunity to alter the negative perception of females, decrease neglect of females and therefore equalise the ratio of males:females. However only two examples of studies were found where population sex ratio was studied in response to an intervention and neither found that the ratio differed significantly following the intervention [43, 51].

#### Impact 4: Reduce risks to public health

In many dog population management interventions the intended beneficiaries are not only dogs but also people and other animals. Risks presented by dogs include zoonotic diseases (e.g. rabies, echinococcosis and leishmaniasis) and bites that may or may not be associated with disease transmission. Given these risks, measuring the impact of dog population management on public health is relatively commonly reported in the literature.

##### Reduction in dog bites

The indicator of **number of dog bites** is an example where the method used to measure the indicator needs to be clearly stated and changes in incidence must be assessed using the same measure. Dog bites can be measured through different methods including through questionnaires, officially reported dog bites (whether dogs bites are reportable will vary with country), emergency room visits for dog bites (part of the previous category but for more serious bites than those only reported to local clinics/GPs), and bites that require surgical reconstruction [53].

Timeframes used to explore bite incidence can also differ, especially where questionnaire surveys are used, ranging from bites occurring throughout the interviewees lifetime [29, 54], or within the previous five [36], two [55] or one [56] year/s.

The only example of using dog bite incidence as an indicator of the impact of an intervention was by Reece et al. [57]. Monthly dog bite incidence data from Jaipur, India, revealed a peak incidence around 10 weeks after the peak whelping date of street dogs [52]. This was attributed to the fact that during the period of whelping puppies become most visible and attractive to people, especially to children, resulting in closer interactions and contacts with puppies and dams. They also found a significant decline in bite incidence over the nearly 15 year period of their intervention, which included spaying the vast majority of female roaming dogs. Such decline was attributed to a reduction in density of roaming dogs and a reduction in maternal aggression over encounters with puppies, as fewer puppies were born.

##### Reduction in rabies risk

Rabies is perhaps the most feared public health risk from dogs. It is an almost invariably fatal viral disease with over 99% of all human cases resulting from infected dog bites [7]. Dog vaccination campaigns aim to reduce or eliminate rabies risk by establishing herd immunity in the dog population. Measuring the impact of a rabies vaccination campaign generally involves the use of three indicators: the (1) **number of suspect/confirmed rabid dog bites or post-exposure prophylaxes (PEP) provided**, (2) **number of dog rabies cases,** and (3) **number of human rabies cases**. Other dog population management activities may be used alongside vaccination, such as dog reproduction control, the combination of reproduction control and vaccination has been shown to reduce rabies risk (e.g. [41]), however an estimate of the contribution that each activity makes to this reduction has not been reported in the literature.

Dog bite incidence can be measured in a number of ways, however for assessing rabies control interventions the **number of dog (or animal) bites treated by health care centres as suspect rabid** has proven useful [57, 58]. Collecting data on suspect rabid dog bites appears relatively simple if this data is reported to a central authority (usually a human health department) and is publically available (e.g. as used by Reece et al. [57]). When the data is not made publically available, access may require approaching health centres directly and asking for their cooperation in collecting and reporting bite data.

The dog bite indicator is also influenced by people’s propensity to report bites for treatment, hence if the intervention includes an education programme on bite treatment or improving the delivery of PEP through health services, incidence of reported bites may increase irrespective of number of bites occurring. For example, in Colombo, Sri Lanka, the number of dog rabies cases decreased over the 5 years of the intervention but the number of bites treated at the general hospital increased along with an improved understanding of the need for PEP as measured through a questionnaire [59]. Hence, it is suggested more than one indicator is used. Cleaveland et al. [58] used dog bites reported by three district hospitals as well as dog rabies cases reported by livestock field officers to evaluate the impact of mass dog vaccination against rabies in Tanzania. Putra et al. [60] used all three indicators in combination (dog bites, dog rabies cases and human rabies cases) to evaluate the impact of a rabies vaccination campaign on Bali, Indonesia.

Using the **number of dog rabies cases** as an indicator can present challenges, because of the difficulties in measuring this parameter. Given the low incidence of rabies, random sampling of animals for a rabies diagnosis, which can only be performed post-mortem, would require huge numbers of animals to be killed in order to confirm a single case; an unethical and practically implausible approach. Surveillance should instead target high-risk animals; those that are biting, behaving strangely, morbid or found dead [61]. Rabies presents with noticeable clinical signs and there is no carrier status (persistently healthy animals that shed virus, although infected dogs may shed virus in the days immediately preceding the onset of clinical signs) and so focus on diagnostic testing of high-risk animals is appropriate. For example, municipal veterinary department records were used successfully to evaluate intervention impact in Colombo, Sri Lanka where this targeted surveillance approach was used [59]. However, detection rates based on laboratory diagnosis of dog rabies cases are notoriously low; this potentially could result in the failure of elimination programmes as control measures are prematurely relaxed. Townsend et al. [61] concluded that at least 5% but ideally 10% of cases need to be detected to have realistic prospects of eliminating rabies.

Given that rabies is often well recognised, reporting of suspected cases on the basis of clinical signs should also be considered as a useful comparative measure of dog rabies incidence. The most detailed estimates of dog rabies incidence have been generated by contact tracing methodology, as has been adopted in Tanzania [62, 63], and which exploits the fact that rabies events are often memorable, which allows the timing of transmission events and cases to be determined retrospectively. Other less intensive methods of measurement that have shown success in detecting suspected dog rabies cases (and dog bites) include involvement of community based ‘rabies workers’ [64]; rabies projects run by school-children [64] and incentives provided to livestock field officers to report suspect rabid dogs [58].

Data on the **number of human rabies deaths** can be obtained in countries where rabies is a reportable disease as this data is usually available from a central government repository, or in some cases, regionally via an intergovernmental organisation (e.g. in the Americas, rabies data is collected and made available online via the Pan-American Health Organisation, PAHO). For example Chomel et al. [65] used rabies case data from the Peruvian Ministry of Health to evaluate the impact of a mass dog vaccination campaign in Lima, Peru; Putra et al. [60] presented human rabies case data collected from the district health authorities in Bali following an island-wide vaccination of dogs; and Belotto et al. [66] reported reductions in human rabies across several Latin American countries following dog vaccination, with the data drawn from the PAHO administered rabies surveillance system. However, in many countries, data from central government records are limited by widespread under-reporting, even when the disease is notifiable. Where rabies is not reportable or is under-recognized, collaborative partnerships with hospitals and local health care service providers are necessary to access this data in order to conduct impact assessment [41, 58].

##### Reduction in risk of Echinococcus granulosus

Human cystic echinococcosis is a disease caused by the tapeworm *Echinococcus granulosus* that leads to hydatid cysts developing in the liver and lungs of people; it can be life-threatening if left untreated. Dogs are primary final hosts in the cycle of *E. granulosus* and sheep are the main intermediate host. Although many other animal species can function as intermediate hosts, the dog-sheep cycle accounts for 95% of human cystic echinococcosis cases [67]. People become infected by ingesting food, water or soil contaminated with stool from infected dogs or during close contact with an infected dog whose fur is contaminated. Dog-related control activities for *E. granulosus* are through regular deworming of dogs with praziquantel and preventing their access to infected offal through inspection and proper disposal of offal from slaughter houses and at home slaughter.

Indicators reported in the literature for assessing the impact of dog interventions included the **number or percentage of sheep with liver or lungs infected with** ***E. granulosus cysts*** **at the time of slaughter**, the **incidence of human cystic echinococcosis** and the **number or percentage of live dogs found to be infected with** ***E. granulosus*** **worms**. For example, in a paper describing the announcement of provisional freedom from hydatid disease in New Zealand, the reduction and final elimination of hydatid cysts in sheep, cattle, and deer at the time of slaughter is presented as evidence of the effectiveness of the New Zealand hydatid control programme, which included deworming of dogs among other activities [68]. In Tasmania, the reduction in incidence of cysts in humans, detected at the time of surgery or at necropsy, alongside a reduction in the number of infected sheep at slaughter and also a reduction in the presence of tape worm in dogs, were presented as evidence of the impact of the first 10 years of hydatid disease control [69]. Methods of measuring indicators of infection in dogs with *E. granulosus* have changed over time. Initially ‘purging’, usually through administration of with arecoline hydrobromide, which causes diarrhoea and expulsion of the worm burden, was used to assess whether a dog was infected in the early stages of most interventions (e.g. as summarised for five countries by Craig & Larrieu [67]). However, purging is both unpleasant and has inherent disease risks as live worms with the potential to re-infect are expelled. Furthermore, it is not suitable for young or pregnant dogs and sometimes causes serious illness or death. In recent years, measurement of *E.granulosus* infection in dogs has been performed through serological testing for the presence of antibodies reflecting immune responses to the antigen or measurement of copro-antigens, produced by the worms themselves and expelled in faeces [70].

##### Reduction in risk of leishmaniasis

Leishmaniasis is a disease of both dogs and humans caused by infection with protozoan parasites of the genus *Leishmania*, most cases occur in Africa, Asia and Latin America. Transmission between people, dogs and other animals is via bites of infected female phlebotomine sandflies. Prevention of human infection can be achieved through: 1) direct intervention by people to reduce sandfly bites, e.g. using bed nets and insect repellent; 2) vector control, e.g. spraying; or 3) reservoir control by using insect repellent impregnated collars or ‘pour-on’ repellents for dogs, keeping dogs indoors during peak sandfly biting times or by reducing the infected dog reservoir through culling, although the efficacy of this last approach is much debated [71]. Several vaccine candidates for dogs are under evaluation, while others have recently become commercially available in Brazil and Europe [72]. Impact assessments of interventions involving dogs have included changes in the incidence of both human and dog infection and disease.

Change in the incidence of **officially reported human leishmaniasis cases** was used as a key indicator when assessing the impact of dog culling [71] and of dog vaccination [73]. Palatnik-de-Sousa et al. [73] also used ELISA tests of dog serum samples (confirmed by an indirect immunofluorescence test) as a measure of dog leishmaniasis infection. Dog infection was also measured through direct agglutination tests (DAT) of serological samples by Mazloumi Gavgani et al. [74] when assessing the impact of insecticide-impregnated dog collars. In the same study, two measures of human infection were used, specifically focusing on children: the leishmanin skin test (LST) conversion and the direct agglutination test (DAT) of finger-prick blood samples. One study, aimed at assessing the impact of a repellent and insecticidal spot-on solution for dogs, used serology (rK39 dipstick) as a first screening tool, followed by parasitological tests to confirm the presence/absence of the parasite, including examination of lymph-node smears and PCR of dermal tissue samples [75]. There were no examples of using incidence of clinical disease in dogs, as less than half of infected dogs show clinical signs and asymptomatic dogs have been found to be a transmission risk.

#### Impact 5: Improve public perception

Improving public perception of dog populations may be particularly attractive to those stakeholders who are concerned with political opinion, but is also beneficial from the perspective of dog welfare on the assumption that a more accepting public may treat roaming dogs with greater tolerance and consideration of their welfare.

Attitude statements were commonly used with either yes/no/don’t know options or Likert scales (5 or 7 levels of agreement from “strongly disagree” to “strongly agree”) which allowed dog-owners or non-owners to state their level of agreement [29, 36, 54, 55, 76]. Only one study attempted to measure change in attitude in response to an intervention; this study in Colombo, Sri Lanka, repeated the same questionnaire five years apart on a cross-section of participants each time [59].

A challenge with attitude statements is to ensure that the statements themselves, or the interviewer, do not lead people to respond in a certain way, reducing the validity of the study. Lunney et al. [55] appeared to include only negative attitude statements in their questionnaire, although they may have been preferentially reporting significant negative statement results and the questionnaire itself may have been more balanced. Lunney et al. [36] seemed to ask just one question about whether “…ownerless dogs from the street caused them problems”, which would arguably not expose any empathetic feelings towards the dogs that you may want to identify in an impact assessment. In contrast, a mix of 18 negative and positive statements, adapted and validated by D Knobel from statements he tested in Tanzania [76], were included in a questionnaire used for impact assessment in Colombo, Sri Lanka [59]. Häsler et al. [59] reported a summative score derived from the responses to 11 of these 18 attitude statements, both positive (e.g. “I like having dogs on my street”) and negative (“street dogs pose a danger to people”). The combination of these questions aimed to measure tolerance or acceptance of dogs; the ‘**summative acceptance score**’ was used as an indicator of change in public perception over time.

Most of these studies used questionnaires to explore perceptions, however participatory research methods may prove to be more efficient and revealing; these methods emphasise involvement of local people in analysing problems and designing solutions, often utilising visualisation techniques and ranking or scoring to draw quantifiable conclusions from group discussions [12]. Häsler et al. [59] used participatory methods to explore people’s perception of the **number and type of problems caused by dogs** and asked people to recall how the situation differed from 5 years previously (pre-intervention) in order to evaluate the impact of intervention. This approach yielded fewer problems relating to dogs being reported in the present as compared to 5 years previously. Unfortunately, this approach had not been used before the intervention so it was not possible to assess the accuracy of the recall. However, even if recall was imperfect, this method revealed that people *perceived* the situation to have improved which was very rewarding for the intervention managers.

Another behaviour potentially reflecting public acceptance of street dogs is **adoption of dogs directly from the street**. This is described in more detail in the section on improving care provided to dogs, but could also be used as an indicator of public acceptance of street dogs.

#### Impact 6: Improve rehoming centre performance

Indicators related to rehoming centre performance could be argued to be a measure of centre effectiveness and therefore related to intervention effort rather than impact on the wider dog population. However, some intervention activities have the potential to feed into a centre’s success, sometimes independently of the actions of the centre itself. For example, reproduction control could reduce unwanted births which could reduce intake or alter the age structure, or an improvement in people’s favourable perceptions of dogs could increase adoptions. Hence a discussion of indicators relating to this impact is included in this review.

The USA-based Asilomer Accords [77] was an initiative to create a reliable and shared indicator of centre performance; the **annual live release rate**. The annual live release rate is expressed as the percentage of total outcomes for shelter animals that are live outcomes (adoptions, outgoing transfers, and return to owner/guardian). The total outcomes include all live outcomes plus euthanasia, but excluding euthanasia requested by owner/guardians or dogs that had died or were lost by the centre. Accompanying guidance provided by the Accords includes a set of principles that proved valuable in maintaining collaboration and consistency within the animal shelter community in reporting their annual live release rate. The guidance not only provides very clear definitions of the data used to estimate these rates, but also practical tools, such as a data gathering forms and a simple equation for estimating the rate itself. Annual live release rates have been used to evaluate the impact of interventions on both individual centres and whole communities comprised of several centres [78].

However for centres that have a ‘no-kill’ policy, annual live release rates will always be near to 100% and hence they require additional indicators. Such indicators will also be useful for centres without 100% live release rate to explore their performance in more detail. For example, **intake rates** split by age category, are an indicator of the size and demography of the unwanted dog population and have been used in evaluation of the impact of spay/neuter campaigns in the US [79].

#### Impact 7: Reduce negative impact of dogs on wildlife

There are several ways that dogs may impact on wildlife. Hughes & Macdonald [1] reviewed 69 papers on interactions between dogs and wildlife and found the main interaction was predation of wildlife by dogs, followed by disease transmission to wildlife. Competition with wild carnivores, hybridization and predation of dogs by wild carnivores were more limited. Here we focus on reported uses of indicators reflecting the first two interactions, predation and disease transmission.

One general indicator of potential dog-wildlife interactions is the **presence of dogs within designated wildlife areas**. Manor & Saltz [80] in Israel recorded any dog sightings whilst surveying for mountain gazelle at water holes, using the proportion of observations in which dogs were sighted as a ‘dog-presence index’.

Predation of wildlife by dogs may be difficult to monitor via direct observation during transects or point surveys as predation is rarely observed. However, as with dog rabies cases, which are similarly rare events, community-based volunteers and wildlife rangers can be asked to report the **number of observed wildlife kills by dogs** to a central organisation. Butler et al. [81] used this method to assess the impact of dogs on wildlife in Zimbabwe, this study was not used for impact assessment as no intervention took place at the time. Using the number of observed wildlife kills by dogs as an indicator alone may not be sufficient for assessment of impact on wildlife, as reported by Hughes & Macdonald [1] pp.349; observed wildlife kills is “unquantified in terms of population impacts. Reporting individual instances of predation gives no indication of the impact on local prey populations and, therefore, whether it is of conservation concern”. Hence additional indicators are required to reflect how the wildlife population is being affected by this predation. An ideal approach could be **monitoring population numbers and structure of wildlife prey** at the same time as monitoring presence of dogs and other predators within designated wildlife areas or number of observed wildlife kills by dogs to see if there is any correlation. For example, the dog-presence index used by Manor & Saltz [80] was found to correlate with surviving kid:female gazelle ratios; a greater number of kids per female was associated with declining dog-presence index.

Rabies and canine distemper viruses are considered major pathogens affecting wildlife, particularly carnivore populations. Their short infection cycles and high mortality rates mean infection cannot be maintained in small endangered wild populations. As the number of animals that succumb to infection increases, the number of new susceptible hosts diminishes, and the infection cannot persist. New infections in wildlife populations can be triggered by contact with more abundant reservoir hosts, for example domestic dogs [82]. Measuring the success of interventions to manage both diseases inevitably requires surveillance of both dogs and wildlife. Therefore, providing an **incidence rate of rabies/CDV in both dogs and susceptible wildlife species** within the same area may be a useful indicator of success of disease intervention programmes involving dogs. Further, detailed analysis of the relationships between incidences in the two populations is recommended [83] to understand the mechanism of transmission between wildlife and dogs as reservoir hosts [82], and inform future disease management plans.

The **proportion of the dog/wildlife population with CDV antibodies** may be a useful indicator to measure through blood testing. However CDV antibodies can remain in animals’ circulation many years after exposure to CDV and so blood sampling may not be a good measure of recent disease incidence. CDV antibody seroprevalence could be used for impact assessment if measured over the long-term and across a range of age groups. For example, when used in the Serengeti, this approach revealed that CDV infection in lions occurred sporadically through the 1970s and 1980s, before reappearing in 1994, suggesting that the virus was not persisting in wildlife during this time. Further analyses of domestic dog serological patterns indicated that infection during the 1994 epidemic was introduced by dogs acting as a reservoir host [82]. Where CDV vaccination of dogs is planned as part of dog population management interventions, seroprevalence for CDV antibodies in dogs would only be useful if unvaccinated dogs are sampled as the vaccination itself would result in a positive blood result. Additional information can be generated through serosurveillance in wildlife along with continued surveillance of active CDV disease in dogs through clinical diagnosis of sick dogs and necropsies. No literature reporting the use of these indicators for assessing the impact of a dog intervention on wildlife disease was found at the time of review.

#### Impact 8: Reduce negative impact of dogs on livestock

No literature could be found measuring the impact of a dog intervention on livestock, however some examples of indicators that could be used for impact assessment were found. Adriani & Bonanni [84] reported data from Merops Veterinaria e Ambiente s.r.l. (the insurance company from which farmers access compensation) for the **number of livestock predation events by dogs** in Italy. Farmers also had to report livestock predation to the local authorities so that attempts could be made to trace the dog owner. However data from the insurance company was more accessible than from the local authorities. Presumably there is centralised reporting of predation by dogs in some countries, in particular where there is government compensation for losses. Where this secondary data source does not exist, questionnaire surveys of farmers may be an alternative. For example, Wang & Macdonald [85] asked farmers living around a wildlife park in Bhutan about predation events, although in this case they did not report losses to dogs, only wild predators. Robel et al. [86] asked recruited sheep producers in Kansas, USA, to telephone a ‘hotline’ when a predation event took place, following which a researcher would promptly visit the farm and conduct a necropsy to identify the predator; leading to domestic dogs being identified as responsible for 24.9% of sheep predation and 19.4% of lambs. Palmer et al. [87] also performed necropsies on dead sheep in Utah, USA, to assess the species of the predator involved. However their method of identifying carcasses for necropsy was more intense; spending several months searching regularly for carcasses on foot and by all-terrain vehicle, focusing on the areas surrounding bed grounds and also following scavenging birds; identifying coyotes, cougars and black bears as predators but not domestic dogs.

Similar to the indicator of presence of dogs within designated wildlife areas, as described under impact 7, the **presence of dogs in livestock areas** could also be measured. Robel et al. [86] interviewed sheep producers, including questions about the number of dogs that they owned and whether they were confined. They also utilised scent stations; sifted earth covering perforated plastic capsules containing coyote urine, and observed visits to these scent stations by both coyotes and domestic dogs. This provided a measure of potential predator abundance that could then be compared to reports of actual predation.

#### Review of study quality

The 26 items of literature that reported a change in one or more impacts following a dog population management intervention were reviewed for five aspects of study design and quality. These included whether the study had included a control group and whether this control group had been randomly assigned, or whether it compared a treatment group at baseline to a post-intervention situation and whether any observed changes had subsequently be tested for statistical significance. Finally the use of observers blinded to the level of treatment when conducting data collection was also reviewed. Table 2 provides a summary of this review by impact, with shading to highlight impacts benefiting from higher quality studies (for a more detailed description of study design for each of the 26 studies, see Table 1).

**Table 2:**
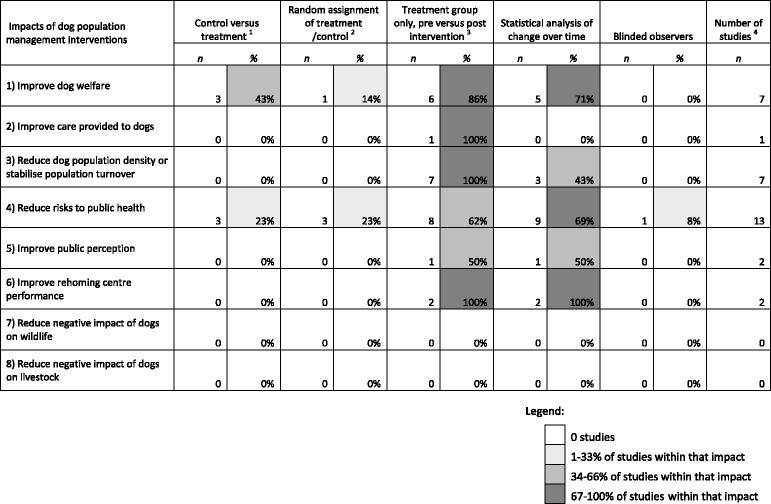
Aspects of study design/quality summarised by impact, shading is used to highlight increasing proportions of the reviewed studies that benefited from aspects of higher study quality

Notes:

^1^Study designs that include a control group; observational, experimental, quasi-experimental and cohort

^2^Experimental study design

^3^Study designs without a control group, but compare changes in a treatment group over time; observational, repeated cross-sectional and longitudinal

^4^The total number of studies exceeds 26 because some studies measured changes in more than one impact

Table 2 illustrates that evaluation of dog population management is in its infancy, and that some impacts are benefiting from higher quality studies than others. The impact of reducing risks to public health through dog population management has received the greatest number of studies, some of which benefited from higher quality aspects of study design. The impacts of an improvement in dog welfare and a reduction in dog density/population stabilisation followed behind reducing risks to public health in terms of study numbers and quality; an increase in studies that used a control and ideally with random assignment would benefit the evidence relating to these impacts. The use of blinded observers was extremely rare in the reviewed studies, such an approach could benefit any study of this type, not least because the observers used were often the same people that conducted the intervention and therefore potentially had a vested interest in a positive result. However, the authors recognise the difficulty in achieving this due to the visibility of dog population management interventions, not least where marking dogs as having been intervened (e.g. ear notches for sterilised or collars for vaccinated) is required for smooth running of the implementation. Our search failed to identify scientific studies quantifying the impact of dog population management on wildlife and livestock, and this highlights the need for future research in this area.

## Conclusion

This scoping review identified a range of indicators that have been used for assessing the impact of dog population management interventions and have the potential for use in future assessments. The fact that these indicators were used and reported in the literature to show a change over time suggests that they are measurable, although some may be more time consuming to measure than others. However this review does not attempt to judge their validity (valid indicators are those that genuinely reflect a change in the desired impact), sensitivity (sensitive indicators reflect small changes in the impact) or their reliability (reliable indicators can be measured by methods that are repeatable and unbiased). Each indicator would benefit from further investigation, including whether relevant methods of measurement have good inter- and intra-observer reliability and how well indicators assumed to be measuring the same impact covary over time and against non-intervention control populations; a potential reflection of their validity and sensitivity.

Due to the limited number of studies available that fulfilled our inclusion criteria, this scoping review included conference presentations, unpublished reports and personal communications. Although use of grey literature increased our potential to find novel and apparently viable methods of measuring indicators of impact, it also risked including studies that were not robust as they had not been scrutinized by peer review. The search was also limited to literature available in English, as the need for dog population management appears greater in the developing world, this may have reduced our ability to benefit from learnings in Latin America, Africa and Asia.

The scarcity of studies that assess the impact of dog population management interventions is remarkable. Further, those studies that do exist do not always benefit from robust study design; often not benefiting from control group(s), statistical analysis of apparent changes or blinded observers during data collection. Dogs are ubiquitous in human societies and most of these expend resources in managing dog populations, whether through a programme of work managed by government or non-governmental organisations or simply through the contribution that individual dog owners make to control their dogs and offspring. Perhaps the widespread and historical nature of these interventions is a partial explanation; many have been in place for several decades and have become ingrained as accepted practice rather than persisting due to evaluation revealing their positive impact. However, there has been a surge in development of new approaches to dog population management in recent years; including low-cost and high throughput spay/neuter clinics launched in the US in the 1970s [88]; piloting and passing legislation to support Animal Birth Control as the national approach to dog population and rabies control in India in 2001; the World Health Organisation’s promotion of mass vaccination of dogs for canine rabies control [89] and recent efforts to develop non-surgical fertility control for dogs and cats [90]. Yet impact assessment still appears relatively rare even amongst these more novel approaches. An additional reason may be that the drivers of these interventions are government or non-governmental organisations conducting this work for public good, as opposed to scientists setting out to test a hypothesis. This goes part way to explain the scarcity of publications and why some may not be of high quality; however it also exposes an opportunity for scientists to apply their expertise in partnership with interventions managers.

Discussions with intervention managers revealed that several challenges prevent them from monitoring and reporting on impact. Resources (staff time and money) are limited and tend to be spent on implementing the intervention instead of monitoring or evaluating its impact. Managers may not know which indicators are most meaningful or cost-effective to monitor in the long-term. Even when monitoring has taken place, data remains unanalysed and unevaluated due to lack of suitable skills, time or money to invest in this essential stage. Where analysis and evaluation has taken place, dissemination of results often may not occur due to lack of time or a perception that this subject may fall outside venues for publications. These discussions indicate opportunities for funders of interventions to provide additional support, access to scientists and encouragement to ensure impact assessment is conducted and disseminated.

As for all impacts, but perhaps best illustrated by the impact of reducing risks to public health, measuring more than one indicator appears to be the most effective way to expose change over time. For example, the combination of suspect rabid dog bites, dog rabies cases and human rabies cases when measuring changes in rabies during interventions appears ideal (e.g. [60]).

The important role that dogs play in almost all human communities, whether as working animals or companions, cannot be denied. Dogs also present risks to human communities and suffer welfare problems themselves, and so efforts to manage their populations to reduce risks and maintain good welfare will always be needed. The design of interventions to manage dog populations will be necessarily varied in response to variation in dog ownership practices, population dynamics and the priority of risks presented at a local level. All interventions should make attempts to measure their effectiveness and conduct regular impact assessments to inform improvements in effectiveness and efficiency. This scoping review indicates that impact assessment of dog population management interventions is currently in its infancy. There are notable examples of innovation and dedication in response to this challenge but there is also substantial room for progress.

Crucially, intervention managers and funders of interventions have a role to play in ensuring conditions are optimal to facilitate impact assessment. By providing the resources and technical guidance to ensure that meaningful indicators are monitored and that data is analysed, evaluated and results and learning shared for the benefit of the wider dog population management community.

## References

[1] Hughes J, Macdonald DW (2013). A review of the interactions between free-roaming domestic dogs and wildlife. Biol Conserv.

[2] Beran GW (1982). Ecology of dogs in the Central Philippines in relation to rabies control efforts. Comp Immunol Microbiol Infect Dis.

[3] De Balogh K, Wandeler A, Meslin F-X (1993). A dog ecology study in an urban and a semi-rural area of Zambia. Onderstepoort J Vet Res.

[4] Davlin SL, Vonville HM (2012). Canine rabies vaccination and domestic dog population characteristics in the developing world: a systematic review. Vaccine.

[5] HSI. U.S. shelter and adoption estimates. 2015. http://www.humanesociety.org/issues/pet_overpopulation/facts/pet_ownership_statistics.html. Accessed November 30, 2015.

[6] Hampson K, Coudeville L, Lembo T, Sambo M, Kieffer A, Attlan M, et al. Estimating the global burden of endemic canine rabies. PLoS Negl Trop Dis. 2015;9 doi:10.1371/journal.pntd.0003709. 10.1371/journal.pntd.0003709 PMC4400070 25881058

[7] WHO. WHO Expert Consultation on Rabies, 2nd report. Techincal Report Series 982 2013. http://www.who.int/rabies/resources/en/. Accessed 30 Nov 2015.

[8] Gilchrist J, Sacks JJ, White D, Kresnow M-J (2008). Dog bites: still a problem?. Inj Prev.

[9] Mays N, Roberts E, Popay J, Fulop N, Allen P, Clarke A, Black N (2001). Synthesising research evidence. Stud. Organ. Deliv. Heal. Serv. Res. Methods.

[10] Coalition ICAM (2015). Are we making a difference? A guide to monitoring and evaluating dog population management interventions.

[11] 1st International Dog Population Management conference. York, UK. 2012. http://www.oie.int/doc/en_document.php?numrec=4178303. Accessed 16 May 2017.

[12] Chambers R. Who Counts? The Quiet Revolution of Participation and Numbers. IDS Research Summary of IDS Working Paper 296. 2007. https://www.ids.ac.uk/idspublication/who-counts-the-quiet-revolution-of-participation-and-numbers1. Accessed 30 Nov 2015.

[13] Sankey C, Häsler B, Hiby E. Change in public perception of roaming dogs in Colombo City. 1st DPM Conf., York, UK. 2012. http://www.oie.int/doc/en_document.php?numrec=4178303. Accessed May 2017.

[14] Steinberger R. A roadmap to creating successful measurable outcomes through high volume spay/neuter in chronic poverty on a Lakota Reservation in the US. 1st DPM Conf., vol. 41, York, UK 2012. Accessible at http://www.oie.int/doc/en_document.php?numrec=4178303. Accessed 16 May 2017.

[15] Yoak AJ, Reece JF, Gehrt SD, Hamilton IM (2014). Disease control through fertility control : secondary benefits of animal birth control in Indian street dogs. Prev. Vet. Med..

[16] Totton SC, Wandeler AI, Ribble CS, Rosatte RC, McEwen SA (2011). Stray dog population health in Jodhpur, India in the wake of an animal birth control (ABC) program. Prev. Vet. Med..

[17] Czupryna AM, Brown JS, Bigambo MA, Whelan CJ, Mehta SD, Santymire RM (2016). Ecology and demography of free-roaming domestic dogs in rural villages near Serengeti National Park in Tanzania. PLoS One.

[18] Laflamme D (1997). Development and validation of a body condition score system for dogs. Canine Pract.

[19] Broom DM. Animal welfare: concepts and measurement. J Anim Sci. 1991:4167–75. 10.2527/1991.69104167x 1778832

[20] Beerda B, Schilder MB, Bernadina W, van Hooff JA, de Vries HW, Mol JA (1999). Chronic stress in dogs subjected to social and spatial restriction. II. Hormonal and immunological responses. Physiol Behav.

[21] Hiby E, Rooney N, Bradshaw J (2006). Behavioural and physiological responses of dogs entering re-homing kennels. Physiol Behav.

[22] Garde E, Pérez G, Vanderstichel R, Dalla Villa P, Serpell J (2016). Effects of surgical and chemical sterilization on the behavior of free-roaming male dogs in Puerto Natales, Chile. Prev. Vet. Med..

[23] Pal SK, Ghosh B, Roy S (1998). Agonistic behaviour of free-ranging dogs (*Canis familiaris*) in relation to season, sex and age. Appl Anim Behav Sci.

[24] Rooney N, Gaines S, Hiby E (2009). A practitioner’s guide to working dog welfare. J Vet Behav Clin Appl Res.

[25] Pal SK (2008). Maturation and development of social behaviour during early ontogeny in free-ranging dog puppies in West Bengal, India. Appl Anim Behav Sci.

[26] Rooney NJ, Cowan S (2011). Training methods and owner–dog interactions: links with dog behaviour and learning ability. Appl Anim Behav Sci.

[27] Lee N (2011). CASE STUDY: dog population management on Koh Tao, Thailand.

[28] Steinberger R (2010). Report on the rosebud Sioux Indian reservation spay / neuter project.

[29] Farnworth M, Blaszak K, Hiby E, Waran N (2012). Incidence of dog bites and public attitudes towards dog care and management in Samoa. Anim Welf.

[30] Acosta-Jamett G, Cleaveland S, Cunningham AA, Bronsvoort BMD (2010). Demography of domestic dogs in rural and urban areas of the Coquimbo region of Chile and implications for disease transmission. Prev. Vet. Med..

[31] Hsu Y, Severinghaus LL, Serpell JA (2003). Dog keeping in Taiwan : its contribution to the problem of free-roaming dogs. J Appl Anim Welf Sci.

[32] Morters MK, Bharadwaj S, Whay HR, Cleaveland S, Damriyasa IM, Wood JLN (2014). Participatory methods for the assessment of the ownership status of free-roaming dogs in Bali, Indonesia, for disease control and animal welfare. Prev. Vet. Med..

[33] van Dijk L, Pritchard JC, Pradhan SK, Wells KL. Sharing the Load: A Guide to Improving the Welfare of Working Animals through Collective Action. Rugby: Practical Action Publishing; 2010.

[34] Gsell AS, Knobel DL, Kazwala RR, Vounatsou P, Zinsstag J (2012). Domestic dog demographic structure and dynamics relevant to rabies control planning in urban areas in Africa: the case of Iringa, Tanzania. BMC Vet Res.

[35] Knobel DL, Laurenson MK, Kazwala RR, Boden LA, Cleaveland S (2008). A cross-sectional study of factors associated with dog ownership in Tanzania. BMC Vet Res.

[36] Lunney M, Fèvre SJS, Stiles E, Ly S, San S, Vong S (2012). Knowledge, attitudes and practices of rabies prevention and dog bite injuries in urban and peri-urban provinces in Cambodia, 2009. Int Health.

[37] Pulczer AS, Jones-Bitton A, Waltner-Toews D, Dewey CE (2013). Owned dog demography in Todos Santos Cuchumatán, Guatemala. Prev. Vet. Med..

[38] Kitala P, McDermott J, Kyule M, Gathuma J, Perry B, Wandeler A (2001). Dog ecology and demography information to support the planning of rabies control in Machakos District, Kenya. Acta Trop.

[39] Morters MK, McKinley TJ, Restif O, Conlan AJK, Cleaveland S, Hampson K (2014). The demography of free-roaming dog populations and applications to disease and population control. J Appl Ecol.

[40] Czupryna A, Faust L, Bigambo M, Brown J, Santymire R. Demography and health of village domestic dogs west of Tanzania, East Africa. 1st DPM Conf., York, UK. 2012. Accessible at http://www.oie.int/doc/en_document.php?numrec=4178303. Accessed 16 May 2017.

[41] Reece JF, Chawla SK (2006). Control of rabies in Jaipur, India, by the sterilisation and vaccination of neighbourhood dogs. Vet. Rec..

[42] Hiby LR. Using clinical data to evaluate an ABC intervention. 1st DPM Conf., York, UK. 2012. Accessible at http://www.oie.int/doc/en_document.php?numrec=4178303. Accessed 16 May 2017.

[43] Totton SC, Wandeler AI, Zinsstag J, Bauch CT, Ribble CS, Rosatte RC (2010). Stray dog population demographics in Jodhpur, India following a population control/rabies vaccination program. Prev. Vet. Med..

[44] Hiby LR, Reece JF, Wright R, Jaisinghani R, Singh B, Hiby EF (2011). A mark-resight survey method to estimate the roaming dog population in three cities in Rajasthan, India. BMC Vet Res.

[45] Kayali U, Mindekem R, Yémadji N, Vounatsou P, Kaninga Y, Ndoutamia AG (2003). Coverage of pilot parenteral vaccination campaign against canine rabies in N’Djaména, Chad. Bull. World Health Organ.

[46] Punjabi GA, Athreya V, Linnell JDC (2012). Using natural marks to estimate free- ranging dog *Canis familiaris* abundance in a MARK-RESIGHT framework in suburban Mumbai, India. Trop Conserv Sci.

[47] Belsare AV, Gompper ME (2013). Assessing demographic and epidemiologic parameters of rural dog populations in India during mass vaccination campaigns. Prev Vet Med.

[48] Beck AM. The ecology of stray dogs: A study of free-ranging urban animals. Purdue University Press, Indiana; n.d. 1973.

[49] Fei SY, Chiang JT, Fei CY, Chou CH, Tung MC (2012). Estimating stray dog populations with the regression method versus Beck’s method: a comparison. Environ Ecol Stat.

[50] Reece JF. Results from a Street Dog and Rabies Control (ABC) Programme in Jaipur, India. 1st DPM Conf., York, UK. 2012. Accessible at http://www.oie.int/doc/en_document.php?numrec=4178303. Accessed 16 May 2017.

[51] Sharma K. Dog population management in Nepal, HART. 1st DPM Conf., York, UK. 2012. Accessible at http://www.oie.int/doc/en_document.php?numrec=4178303. Accessed 16 May 2017.

[52] Reece JF, Chawla SK, Hiby EF, Hiby LR (2008). Fecundity and longevity of roaming dogs in Jaipur, India. BMC Vet Res.

[53] Rowan AN. Dog Bites as an Index of Dog Population Management. 1st DPM Conf., York, UK. 2012. Accessible at http://www.oie.int/doc/en_document.php?numrec=4178303. Accessed 16 May 2017.

[54] Pérez GE, Garde EJ. Human attitudes toward dogs in a semi-rural community in south- central Chile. 1st DPM Conf., York, UK. 2012. Accessible at http://www.oie.int/doc/en_document.php?numrec=4178303. Accessed 16 May 2017.

[55] Lunney M, Jones A, Stiles E, Waltner-Toews D (2011). Assessing human-dog conflicts in Todos Santos, Guatemala: bite incidences and public perception. Prev. Vet. Med..

[56] Hergert M, Nel LH (2013). Dog bite histories and response to incidents in canine rabies-enzootic KwaZulu-Natal. South Africa PLoS Negl Trop Dis.

[57] Reece JF, Chawla SK, Hiby AR (2013). Decline in human dog-bite cases during a street dog sterilisation programme in Jaipur, India. Vet Rec.

[58] Cleaveland S, Kaare M, Tiringa P, Mlengeya T, Barrat J (2003). A dog rabies vaccination campaign in rural Africa: impact on the incidence of dog rabies and human dog-bite injuries. Vaccine.

[59] Häsler B, Hiby E, Gilbert W, Obeyesekere N, Bennani H, Rushton J (2014). A one health framework for the evaluation of rabies control Programmes: a case Study from Colombo City, Sri Lanka. PLoS Negl Trop Dis.

[60] Putra AAG, Hampson K, Girardi J, Hiby E, Knobel D, Mardiana IW (2013). Response to a rabies epidemic Bali, Indonesia 2008-2011. Emerg Infect Dis.

[61] Townsend SE, Lembo T, Cleaveland S, Meslin FX, Miranda ME, Putra AAG (2013). Surveillance guidelines for disease elimination: a case study of canine rabies. Comp Immunol Microbiol Infect Dis.

[62] Hampson K, Dobson A, Kaare M, Dushoff J, Magoto M, Sindoya E (2008). Rabies exposures, post-exposure prophylaxis and deaths in a region of endemic canine rabies. PLoS Negl Trop Dis.

[63] Hampson K, Dushoff J, Cleaveland S, Haydon DT, Kaare M, Packer C (2009). Transmission dynamics and prospects for the elimination of canine rabies. PLoS Biol.

[64] Kitala PM, McDermott JJ, Kyule MN, Gathuma JM (2000). Community-based active surveillance for rabies in Machakos District, Kenya. Prev. Vet. Med..

[65] Chomel B, Chappuis G, Bullon F, Cardenas E, David de Beublain T, Lombard M, et al Mass vaccination campaign against Rabies : are dogs correctly Protected ? The Peruvian experience. Rev Infect Dis 1988;10:S697–S702. 10.1093/clinids/10.supplement_4.s697 3206083

[66] Belotto A, Leanes LF, Schneider MC, Tamayo H, Correa E (2005). Overview of rabies in the Americas. Virus Res.

[67] Craig PS, Larrieu E (2006). Control of cystic echinococcosis/hydatidosis: 1863-2002. Adv Parasitol.

[68] Pharo H (2002). New Zealand declares “ provisional freedom ” from hydatids. Theatr Surv.

[69] Beard TC (1978). Evidence that a hydatid cyst is seldom “as old as the patient.”. Lancet.

[70] Reichel M, Baber D, Craig P, Gasser R (1996). Cystic echinococcosis in the Falkland Islands. Prev Vet Med.

[71] Nunes CM, Pires MM, da Silva KM, Assis FD, Gonçalves Filho J, Perri SHV (2010). Relationship between dog culling and incidence of human visceral leishmaniasis in an endemic area. Vet Parasitol.

[72] Otranto D, Dantas-Torres F (2013). The prevention of canine leishmaniasis and its impact on public health. Trends Parasitol.

[73] Palatnik-de-Sousa CB, Silva-Antunes I, Morgado ADA, Menz I, Palatnik M, Lavor C (2009). Decrease of the incidence of human and canine visceral leishmaniasis after dog vaccination with Leishmune in Brazilian endemic areas. Vaccine.

[74] Mazloumi Gavgani AS, Hodjati MH, Mohite H, Davies CR (2002). Effect of insecticide-impregnated dog collars on incidence of zoonotic visceral leishmaniasis in Iranian children: a matched-cluster randomised trial. Lancet.

[75] Otranto D, Paradies P, Lia RP, Latrofa MS, Testini G, Cantacessi C (2007). Efficacy of a combination of 10% imidacloprid/50% permethrin for the prevention of leishmaniasis in kennelled dogs in an endemic area. Vet Parasitol.

[76] Knobel DL, Laurenson KM, Kazwala RR, Cleaveland S (2008). Development of an item scale to assess attitudes towards domestic dogs in the United Republic of Tanzania. Anthrozoos a Multidiscip. J Interact People Anim.

[77] Anon. The Asilomar Accords. Asilomar, Pacific Grove, California. Accessible at https://www.shelteranimalscount.org/who-we-are/history. 2004.

[78] Weiss E, Patronek G, Slater M, Garrison L, Medicus K (2013). Community partnering as a tool for improving live release rate in animal shelters in the United States. J Appl Anim Welf Sci.

[79] Frank JM, Carlisle-Frank PL (2007). Analysis of programs to reduce overpopulation of companion animals: do adoption and low-cost spay/neuter programs merely cause substitution of sources?. Ecol Econ.

[80] Manor R, Saltz D (2004). The impact of free-roaming dogs on gazelle kid/female ratio in a fragmented area. Biol Conserv.

[81] Butler J, du Toit J, Bingham J (2004). Free-ranging domestic dogs (*Canis familiaris*) as predators and prey in rural Zimbabwe: threats of competition and disease to large wild carnivores. Biol Conserv.

[82] Cleaveland S, Mlengeya T, Kaare M, Haydon D, Lembo T, Laurenson MK (2007). The conservation relevance of epidemiological research into carnivore viral diseases in the serengeti. Conserv Biol.

[83] Woodroffe R (1999). Managing disease threats to wild mammals. Anim Conserv.

[84] Adriani S, Bonanni M. Stray dogs and damage to sheep farms in the Oristano Province, Sardinia, Italy. 1st DPM Conf., York, UK. 2012. Accessible at http://www.oie.int/doc/en_document.php?numrec=4178303. Accessed 16 May 2017.

[85] Wang SW, Macdonald DW (2006). Livestock predation by carnivores in Jigme Singye Wangchuck National Park. Bhutan Biol Conserv.

[86] Robel RJ, Dayton AD, Henderson FR, Meduna RL, Spaeth CW (1981). Relationships between husbandry methods and sheep losses to canine predators. J Wildl Manag.

[87] Palmer BC, Conover MR, Frey SN (2010). Replication of a 1970s study on domestic sheep losses to predators on Utah’s summer rangelands. Rangel Ecol Manag.

[88] Rowan AN, Williams J (1987). The success of companion animal management programs: a review. Anthrozoös.

[89] WHO (2004). WHO technical report series 931. WHO expert consultation on rabies.

[90] Alliance for the Contraception of Cats & Dogs. Contraception and Fertility Control in Dogs and Cats. Portland: ACC&D. 2013. Accessible at http://www.acc-d.org/resource-Library/e-Book.

